# Temperature-driven massless Kane fermions in HgCdTe crystals

**DOI:** 10.1038/ncomms12576

**Published:** 2016-08-30

**Authors:** F. Teppe, M. Marcinkiewicz, S. S. Krishtopenko, S. Ruffenach, C. Consejo, A. M. Kadykov, W. Desrat, D. But, W. Knap, J. Ludwig, S. Moon, D. Smirnov, M. Orlita, Z. Jiang, S. V. Morozov, V.I. Gavrilenko, N. N. Mikhailov, S. A. Dvoretskii

**Affiliations:** 1 Laboratoire Charles Coulomb, UMR CNRS 5221, University of Montpellier, Montpellier 34095, France; 2 Institute for Physics of Microstructures, Russian Academy of Sciences, Nizhny, 603950 GSP-105 Novgorod, Russia; 3 Institute of High Pressure Institute Physics, Polish Academy of Sciences, 01-447 Warsaw, Poland; 4 National High Magnetic Field Laboratory, Tallahassee, Florida 32310, USA; 5 Department of Physics, Florida State University, Tallahassee, Florida 32306, USA; 6 Laboratoire National des Champs Magnétiques Intenses, CNRS-UJF-UPS-INSA, 38042 Grenoble, France; 7 Faculty of Mathematics and Physics, Charles University, Ke Karlovu 5, 121 16 Prague 2, Czech Republic; 8 School of Physics, Georgia Institute of Technology, Atlanta, Georgia 30332, USA; 9 Lobachevsky State University of Nizhny Novgorod, Nizhny, 603950 Novgorod, Russia; 10 Institute of Semiconductor Physics, Siberian Branch, Russian Academy of Sciences, pr. Akademika Lavrent'eva 13, 630090 Novosibirsk, Russia; 11 Novosibirsk State University, 630090 Novosibirsk, Russia

## Abstract

It has recently been shown that electronic states in bulk gapless HgCdTe offer another realization of pseudo-relativistic three-dimensional particles in condensed matter systems. These single valley relativistic states, massless Kane fermions, cannot be described by any other relativistic particles. Furthermore, the HgCdTe band structure can be continuously tailored by modifying cadmium content or temperature. At critical concentration or temperature, the bandgap collapses as the system undergoes a semimetal-to-semiconductor topological phase transition between the inverted and normal alignments. Here, using far-infrared magneto-spectroscopy we explore the continuous evolution of band structure of bulk HgCdTe as temperature is tuned across the topological phase transition. We demonstrate that the rest mass of Kane fermions changes sign at critical temperature, whereas their velocity remains constant. The velocity universal value of (1.07±0.05) × 10^6^ m s^−1^ remains valid in a broad range of temperatures and Cd concentrations, indicating a striking universality of the pseudo-relativistic description of the Kane fermions in HgCdTe.

## Abstract (web summary)

Kane fermions are predicted to be tunable with external parameters such as temperature. Here, Teppe *et al*. show a band structure evolution of bulk HgCdTe as temperature is tuned across topological phase transition, demonstrating that Kane fermions change sign in rest-mass and remain constant in velocity.

## Graphical abstract

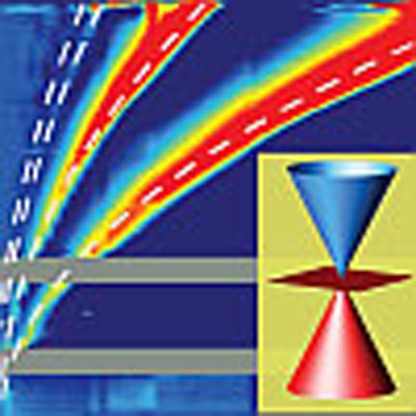

In condensed matter systems, the interaction of electrons with a periodic crystal lattice potential can give rise to low-energy quasiparticles that mimic the relativistic dynamics of Dirac particles in high-energy physics. Perhaps the most spectacular demonstration of this concept was given ten years ago by the isolation of a monolayer of carbon atoms forming a graphene^1^ sheet. The electrons in graphene behave as two-dimensional (2D) massless fermions with gapless conical bands that obey the Dirac equation. Subsequently, further condensed matter analogues of high-energy relativistic fermions were demonstrated such as edge or surface states of 2D or three-dimesnional (3D) topological insulators^2^,^3^,^4^ and 3D Dirac semimetals with linear energy-momentum dispersion in all three momentum directions^5^,^6^,^7^.

Recently, another massless Dirac-like quasiparticle has been discovered in Hg_1−*x*_Cd_*x*_Te at an inverted-to-normal band structure topological transition existing at the critical cadmium concentration^8^ *x*_C_≈0.17. These 3D massless Kane fermions are not equivalent to any other known relativistic particles. Similar to the pseudospin-1 Dirac–Weyl system^9^, their energy dispersion relation features cones crossed at the vertex by an additional flat band. The bandgap and the electronic dispersion in Hg_1−*x*_Cd_*x*_Te can be tuned intrinsically by adjusting the chemical composition, or externally, by changing temperature^10^. The ability to control the properties of quasiparticles with relativistic behaviour in a tabletop condensed-matter experiment holds vast scientific and technological potential. However, the variation of the chemical composition in Hg_1−*x*_Cd_*x*_Te crystals does not allow for fine-tuning of the bandgap in the vicinity of the phase transition due to inherent fluctuations of Cd concentration. Contrarily, a temperature-driven evolution of the band structure (see also Supplementary Note 1) provides a conceptually straightforward, yet very accurate and detailed probe of the relativistic properties of Kane fermions, while tuning the 3D Hg_1−*x*_Cd_*x*_Te across the gapless state at the topological transition between a normal state and an inverted bandgap state. The appearance of a non-zero gap does not exclude relativistic properties of the Kane fermions in Hg_1−*x*_Cd_*x*_Te, which is retained at energies significantly above the gap. The energy dispersion asymptotically tends to a linear behaviour, as it is for relativistic electrons at high energies. The cutoff energies for relativistic behaviour in the Hg_1−*x*_Cd_*x*_Te compounds and in other 3D Dirac–Weyl semimetals are related to the presence of high-lying conduction bands and low-lying valence bands.

Here we conduct a systematic optical investigation of the dispersion of Kane fermions in Hg_1−*x*_Cd_*x*_Te crystal as a function of temperature and magnetic field. From the experimental point of view, the use of temperature as a band structure tuning parameter in magneto-optical studies is challenging and demands ultimate quality samples. This is because the observation of well-defined optical resonances requires high carrier mobility, which degrades with increasing temperature due to the increase of scattering on phonons. Our bulk Hg_1−*x*_Cd_*x*_Te samples were grown by molecular beam epitaxy. The Cd concentration was chosen to enable exploring the bandgap *E*_g_(*x*, *T*) space across the semimetal-to-semiconductor phase transition using temperature for fine gap-at-will tuning. The sample A, *x*=0.175, is a standard narrow-gap semiconductor at any temperature. The sample B, *x*=0.155, is a semimetal at low temperatures with a negative bandgap corresponding to the inverted band order. As the temperature increases, the inverted bandgap closes as the system undergoes a semimetal-to-semiconductor phase transition at the critical temperature *T*_C_≈77 K followed by the opening of a gap in the normal state. Based on a simplified Kane model, we determine the Kane fermions velocity and rest mass. The rest mass experiences a change of sign at the critical temperature of topological phase transition. Our study reveals a universal velocity of 1.07 × 10^6^ m s^−1^ in HgTe crystals. This further allows to determine the Kane fermion rest mass from all experimental results, past or future, close to the phase transition.

## Results

### Band structure evolution with temperature

To describe the electronic structure near the centre of the Brillouin zone in Hg_1−*x*_Cd_*x*_Te close to *x*_C_, we employ a simplified Kane model^11^,^12^ taking into account *k·p* interaction between the Γ_6_ and Γ_8_ bands, while neglecting the influence of the split-off Γ_7_ band. The corresponding (6 × 6) Hamiltonian formally resembles the one for relativistic 3D Dirac particles (see ‘Simplified Kane model' in Methods). By neglecting small quadratic in momentum terms, the eigenvalues of this Hamiltonian can be presented in the form:


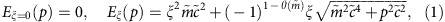


The first eigenvalue *ξ*=0 corresponds to the flat heavy-hole band. The two other eigenvalues describe the electron (*ξ*=+1) and light-hole (*ξ*=−1) conical bands separated by 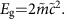 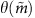 is a Heaviside step function, which equals to 1 for 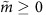 and to 0 if 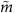 is negative. This representation of Kane fermions in Hg_1−*x*_Cd_*x*_Te contains only two parameters, the rest -mass 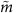 and the universal velocity 
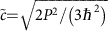
, whereas the material properties are introduced through the Kane's matrix element *P* and the Hg_1−*x*_Cd_*x*_Te bandgap *E*_g_.

**Figure 1:**
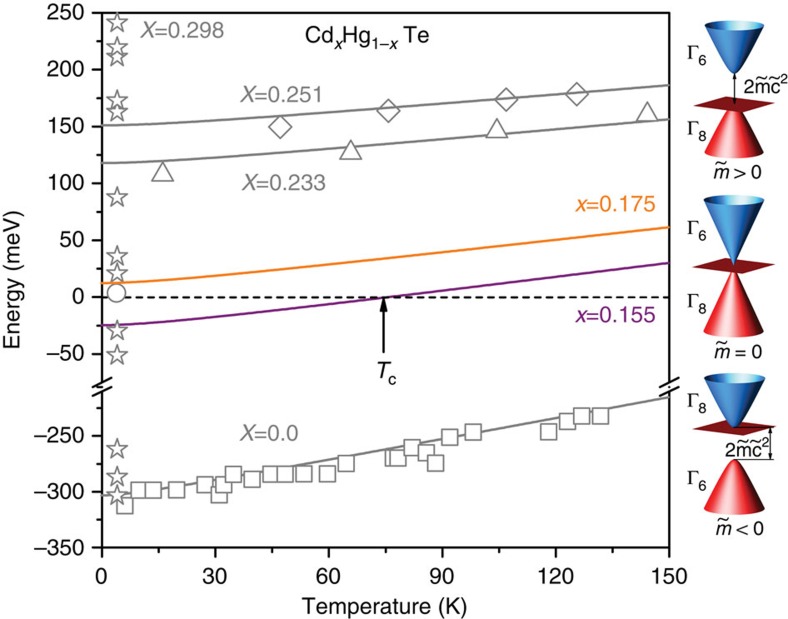
Temperature tuning of the band structure in 3D Hg_*x*_Cd_1−*x*_Te. (**a**) The energy bandgap in Hg_*x*_Cd_1−*x*_Te, defined as the difference between the Γ_6_ and Γ_8_ band extrema at the centre of the Brillouin zone, increases monotonically with temperature and Cd content. Open symbols correspond to experimental data, with circle from ref. 8, squares from ref. 21, stars from ref. 23, and triangles and diamonds from ref. 24, for several Cd concentrations, and the lines show the *E*_g_(*T*) evolution calculated using Supplementary equation (1)^8^ (in Supplementary Note 1). The purple and orange lines correspond to the Cd concentration for samples studied in this work, *x*=0.155 and *x*=0.175. At the critical temperature, *T*_C_, the system undergoes a semimetal-to-semiconductor topological phase transition between inverted and normal states. For *x*=0.155, the gapless state is realized at 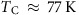. (**b**) Schematic band structure of Kane fermions in 3D Hg_*x*_Cd_1−*x*_Te. As the rest mass of Kane Fermions changes, the electronic dispersion evolves from a standard gapped semiconductor for 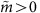 into a semimetal at 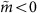. At the point of the topological transition, 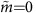, the conical conduction (blue) and the light-hole valence (red) bands are crossed at the vertex by a flat heavy-hole band (brown).

The evolution of Kane fermions in Hg_1−*x*_Cd_*x*_Te is illustrated in Fig. 1. If the rest mass 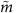 is positive, the crystal is a typical narrow-gap semiconductor with the *s*-type Γ_6_ band lying above the *p*-type Γ_8_ bands, as schematically shown on Fig. 1a. On the other hand, if 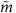
<0, the band order is inverted: the Γ_6_ band lies below the Γ_8_ bands. As the two Γ_8_ bands always touch each other at the Γ point of the Brillouin zone, the band structure is gapless and the crystal is a semimetal. The two distinct phases with different sign of the rest mass are not topologically equivalent, as characterized by a *Z*_2_ topological invariant^13^.

Experimentally, the dispersion of gapless or gapped 2D or 3D Dirac fermions can be conveniently probed through the magnetic field dependence of inter-Landau level transitions^14^,^15^,^16^,^17^,^18^,^19^,^20^. The application of a quantizing magnetic field *B* transforms the zero-field continuum of states into a set of unequally spaced Landau levels (LLs) with a distinct 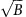 behaviour. In pristine gapless graphene, for example, the LLs have a simple structure given by^14^: 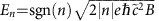, where *ħ* is the Planck constant and *e* is the electron charge. The integer LL index *n* labels electron- (*n*>0) and hole-like (*n*<0) states, and unconventional, zero-energy field-independent *n*=0 LL states. In graphene/boron-nitride heterostructures with zero crystallographic alignment angle, an intrinsic gap Δ opens up separating zeroth dispersionless LLs, *E*_*n*=0_=±Δ/2. Other |*n*|>0 electron(hole)-like LLs shift up(down) by Δ/2 as well: 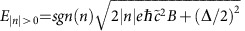 (after ref. 17).

The LL spectrum of massive or massless fermions in Hg_1−*x*_Cd_*x*_Te has a more complex form:


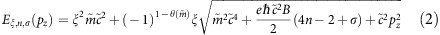


Here, the LL index *n* runs over positive integers *n*=1,2, …. for the states in the electron and light-hole bands (*ξ*=±1). For the zero energy, flat heavy-hole band (*ξ*=0), *n* runs over all non-negative integers, except 1: *n*=0, 2, 3…. The quantum number *σ* accounts for the Kramer's degeneracy lifted by the magnetic field **B**=(0, 0, *B*). The corresponding splitting can be viewed as the Zeeman (spin) splitting of LLs: *E*_*ξ*,*n*,↑_(*p*_*z*_)−*E*_*ξ*,*n*,↓_(*p*_*z*_). The non-parabolicity of the bands implies a strong dependence of this spin-splitting on *p*_*z*_, *B* and on LL and band indices.

### Temperature-induced bandgap opening in narrow gap HgCdTe

**Figure 2:**
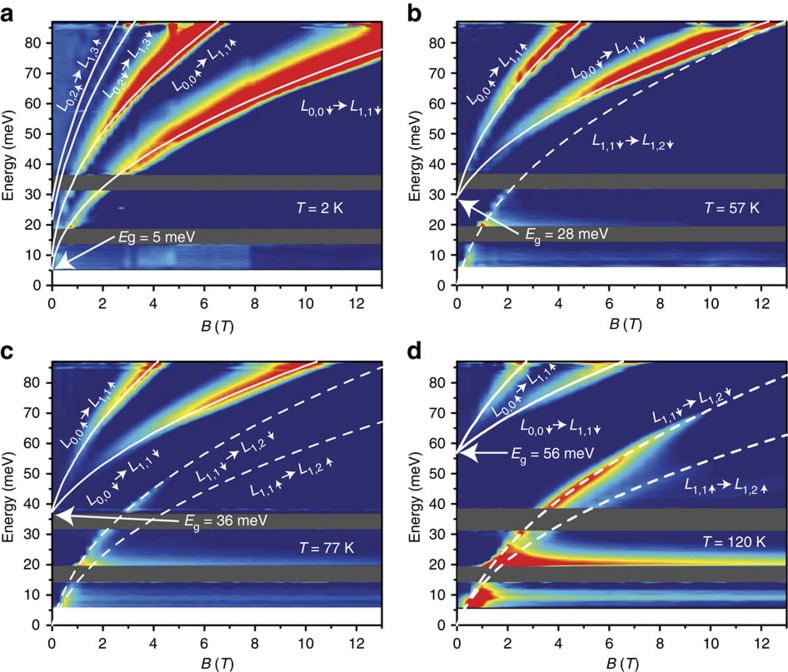
Relative change of absorbance with magnetic field in sample A. False colour maps present inter-LL transitions as a function of magnetic field. White lines represent the fits using the simplified Kane model, allowing for determination of 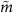 and 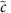. Solid lines correspond to inter-band transitions, whereas dashed lines account for intra-band transitions. The bandgap values at different temperatures, determined by the inter-band transition energies at zero magnetic field, are depicted with arrows. Shaded areas represent the Reststrahlen bands (between 15–20 meV for HgTe/Cd_*x*_Hg_1−*x*_Te band and 32–37 meV for GaAs substrate band). (**a**) At 2 K, only inter-band transitions are seen and a 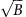-like behaviour of inter-LL resonances and spin splitting of LLs is observed. (**b**) At 57 K, intra-band transitions become visible in addition to previous lines. (**c**,**d**) At higher temperatures the energy difference between inter-band and intra-band lines at zero field clearly increases, corresponding to a gap opening as a function of temperature. The horizontal line observed at 21 meV in **c**,**d** corresponds to the energy of TO CdTe-like phonons, arising in magnetoabsorption due to the electron–phonon interaction. As discussed in refs 27, 28, the frequency of such phonon mode in HgCdTe alloys is almost independent on temperature. In addition, the horizontal line observed at 9 meV in **c**,**d** is attributed to optical transitions from impurities as discussed in refs 29, 30. Indeed, because of weak Hg–Te bonds, mercury vacancies are always present in HgCdTe alloys, even in high-quality *n*-type materials. The amplitude of these lines rises as temperature increases. It has to be noted that thermal energy at 120 K is 10 meV; thus, any features visible below that energy should not be considered as relevant.

Besides the linear behaviour of the absorption coefficient at zero magnetic field measured in both samples (see Supplementary Fig. 1 and Supplementary Note 2), the presence of pseudo-relativistic 3D fermions with 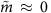 is established at 2 K in the sample A by 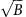-like dependence of optical transitions and a spin splitting of LLs seen in Fig. 2a. The fitting of the two main lines based on equation (2) shows that they are related to the inter-band transitions between the heavy-hole band remaining at zero energy (*ξ*=0) and the *n*=1 spin-up and spin-down LLs with *ξ*=1 (for details, see Supplementary Figs 2 and 3). The extracted bandgap value at 2 K equals 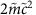
=(5±2) meV. Magneto-optical results obtained at temperatures from 57 to 120 K are shown in Fig. 2b–d. The bandgap, visualized by intersect of the inter-band transitions with the energy axis (shown by white arrows), increases with temperature (see also Supplementary Fig. 4). In addition to inter-band transitions (*Δξ*=1), intra-band LLs transitions (*Δξ*=0) are also observed and fitted with equation (2).

### Temperature-driven phase transition

**Figure 3:**
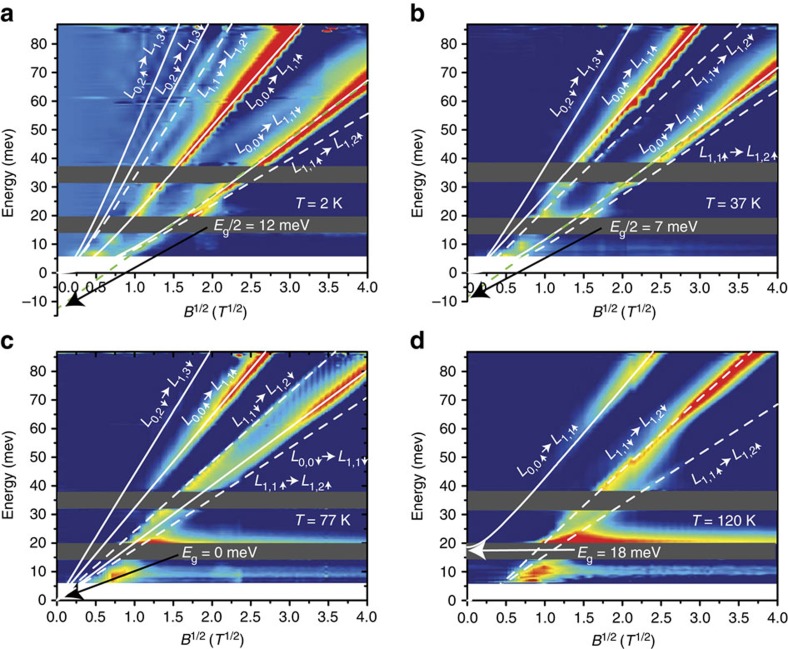
Relative change of absorbance with magnetic field for sample B. Colour maps of the inter-LL transitions as a function of magnetic field in the sample B at temperatures from 2 to 120 K in a square root scale. White lines represent the fits using the simplified Kane model. Solid lines represent inter-band optical transitions, whereas dashed lines correspond to intra-band transitions. Green dotted lines are guides for the eyes accounting for half the bandgap energy at each temperature (with arrows indicating half the bandgap values). (**a**,**b**) At 2 and 37 K, some discrepancies to the 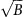 behaviour of inter-LL optical transitions are observed, corresponding to the existence of a negative bandgap. (**c**) At 77 K, a pure 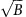 behaviour corresponds to the gapless state and the presence of genuine massless Kane fermions. (**d**) At 120 K, a positive gap opens as seen with the presence of an inter-band optical transition. Shaded areas denote the Reststrahlen bands (between 15–20 meV for HgTe/Cd_*x*_Hg_1−*x*_Te band and 32–37 meV 150 for GaAs substrate band). The horizontal line at 21 meV in **c**,**d** is related to the energy of TO CdTe-like phonons, arising in magnetoabsorbance due to the electron–phonon interaction. The line at 9 meV is attributed to optical transitions from impurities.

**Figure 4:**
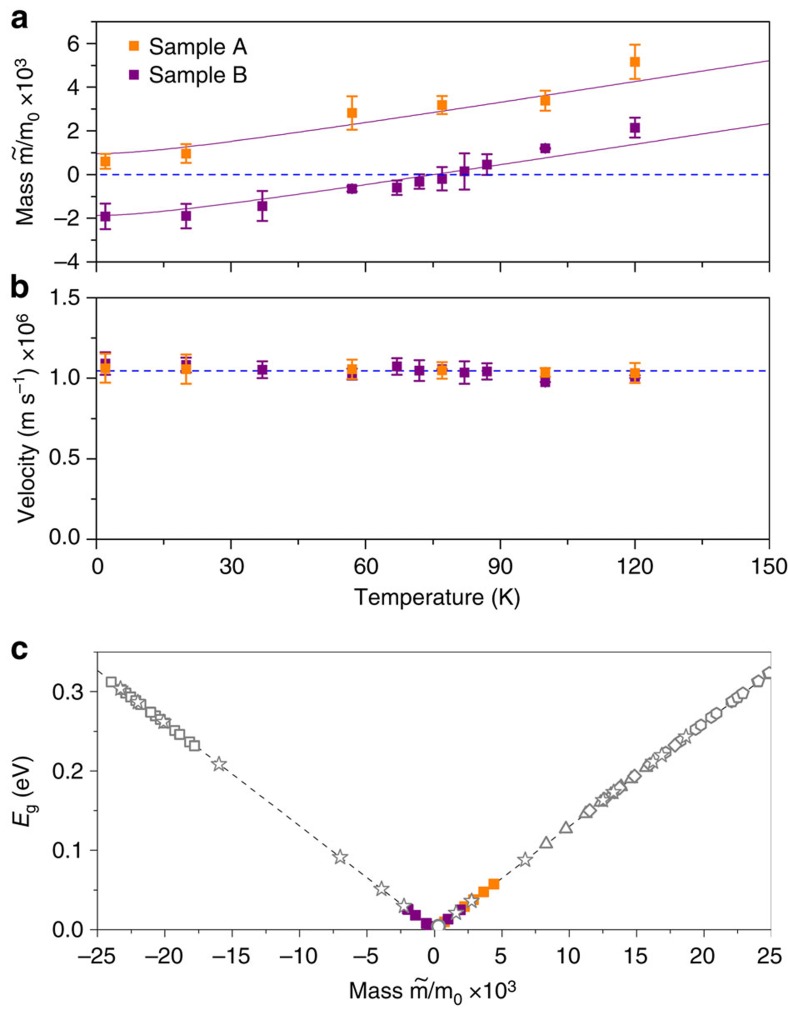
Kane fermion parameters. Orange and purple points correspond to experimental results obtained in this work, whereas open symbols are experimental points from refs 21, 23, 24. (**a**) Rest mass values for both samples are determined by fitting magneto-optical data with the simplified model, including the Γ_6_ and Γ_8_ bands only. Kane fermion rest mass in the sample A is close to zero at low temperatures and increases with temperature up to 120 K. In the sample B, the Kane particle rest mass is negative at low temperatures, it vanishes at 77 K and then becomes positive above *T*_c_. The lines are theoretical curves calculated using Supplementary equation (1)^21^ (in Supplementary Note 1). (**b**) The velocity is nearly constant in both samples between 2 and 120 K. The blue dashed line represents the theoretical value. (**c**) The universal evolution of the bandgap energy versus the Kane fermion rest mass in HgCdTe alloys is shown. The black dashed line serves as a guide for the eyes. The error bars were estimated by calculating the s.d. of the set of parameters obtained from all fittings at given temperature.

Magneto-absorption of the sample B at different temperatures is presented in Fig. 3, in a 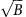-scale for the sake of clarity. It is seen that at low temperatures and low magnetic field values, the LLs transitions exhibit some discrepancies compared with a pure 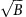 behaviour. Indeed, as shown in Fig. 1, the linear energy dispersion in conduction and valence bands arises in gapless samples only. However, even in the case of small negative (or positive) gap, the bands could be considered as parabolic in the vicinity of the Γ point. It corresponds to the small values of parameter 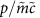, for non-zero rest mass values. At low magnetic fields, the band parabolicity results in a linear behaviour of the LLs transitions as a function of *B*. Therefore, only a precise 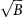 behaviour down to the lowest applied magnetic fields implies a system with genuine massless particles. At temperatures below *T*_C_, the deviation from a pure 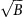 behaviour is well reproduced by the theory and gives absolute values of the rest mass 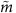 approaching to zero when temperature increases. As seen in equation (2), linear extrapolation of optical transitions in the square-root scale intersects the energy axis at 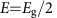, as represented by arrows in Fig. 3a,b. An accurate 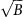 behaviour for all optical transitions is obtained as an evident proof of gap closing at 77 K (Fig. 3c, see also Supplementary Fig. 5). Above the critical temperature, the difference in energies of inter- and intra-band transitions in low magnetic fields becomes visible, as it is for the sample A, meaning that a positive gap between the Γ_6_ and Γ_8_ bands opens. This allows us claiming that at *T*_C_=77 K, a temperature-driven topological phase transition with pseudo-relativistic massless Kane fermions occurs. This fact is highlighted by the change of a sign of particle rest mass seen in Fig. 4a.

### Universal velocity and rest mass description

The rest mass extracted from magneto-optical data and its dependence on temperature is compared with theoretical values using Supplementary equation (1)^21^ (in Supplementary Note 1). In the sample A, the rest mass is positive and increases in the whole range of temperatures, as it is shown in Fig. 4a. As discussed above, in the sample B the Kane fermion rest mass experiences a change of sign corresponding to the temperature-induced semimetal-to-semiconductor topological phase transition, which occurs at 77 K. Supplementary equation (1)^21^ (in Supplementary Note 1) describes very well the experimental rest mass curves for both samples and clearly reproduces the phase transition at 77 K in the sample B. The conic dispersion relation of the massless particles can be therefore realized for the specific range of crystal chemical composition and an according temperature—conditions that provide that the bulk bandgap is fully closed. Interestingly, the Kane fermion velocity 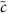 is nearly constant over the whole range of temperatures for both samples with Cd contents of 0.155 and 0.175. The extracted value of 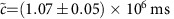
^−1^ is in a very good agreement with the theoretical value defined by 
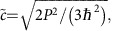
which equals to 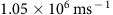 for the well-accepted value of *E*_P_=2*m*_0_*P*^2^/*ħ*^2^≈18.8 eV (ref. 22). Therefore, this universal value of 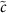 allows for determination of the particle rest mass for bandgap values in the vicinity of the semimetal-to-semiconductor phase transition induced by temperature, Cd content or other external parameter (for example, pressure). Figure 4c shows the variation of the experimental bandgap energy obtained in this work (full blue and red points) and from previous studies^21^,^23^,^24^ (open symbols), as a function of the rest mass, using 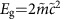 and the universal value of 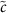.

## Discussion

There are two points limiting the applicability of the simplified Kane model, considering the Γ_6_ and Γ_8_ band only, for actual HgCdTe crystals. The first one, already mentioned above, is related to the existence of other bands, considered as remote and not included in the model. The energy gap between the second and the lowest conduction bands in CdTe exceeds 4 eV, while the corresponding gap in HgTe is about 3 eV (ref. 25). Therefore, the cutoff energies for conduction bands in the simplified model should be lower than 3 eV. For the valence band, the cutoff energy is defined by the energy difference *Δ*≈1 eV between the split-off Γ_7_ band and the heavy-hole band. The second limitation is attributed to the flat heavy-hole band, characterized by an infinite effective mass in the model. To ignore the parabolic terms in the electron dispersion of the heavy-hole band, one has to consider sufficiently low energies *E*, such that the relativistic mass of the fermions 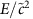 should be significantly lower than the heavy-hole mass *m*_hh_. Assuming *m*_hh_≈0.5*m*_0_, where *m*_0_ is the free electron mass, we arrive at a cutoff energy of ∼3 eV for the flat band approximation, which exceeds *Δ*.

By using temperature as a fine-tuning external parameter we measured the bandgap energy of HgCdTe bulk crystals with well-chosen chemical composition in the vicinity of the semimetal-to-semiconductor phase transition. We clearly observed and accurately measured increasing of the bandgap with temperature in sample A. We also observed genuine massless Kane fermions at the critical temperature of 77 K in sample B. We used the simplified Kane model to determine the pseudo-relativistic Kane fermion parameters 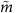 and 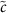 as a function of temperature and Cd content. We observed a change of sign of 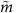 accounting for the temperature-driven topological phase transition. Our results also reveal universal velocity in HgCdTe crystals allowing for the determination of the Kane fermion rest mass from all experimental results in the literature obtained in the vicinity of the phase transition.

## Methods

### Experimental details

We performed magneto-optical studies on two [013]-oriented Hg_1−*x*_Cd_*x*_Te layers, with different Cd concentrations, *x*=0.17 and 0.155. Both films were sufficiently thick (≈3.2 μm) to be considered as 3D materials and thin enough to be transparent in the far-infrared spectral range. The samples were grown by molecular beam epitaxy on semi-insulating GaAs substrates with relaxed CdTe buffers^26^. We used a special ultra-high vacuum multi-chamber molecular beam epitaxy set, which allows for the growth of very high-quality HgCdTe crystals monitored by *in situ* reflection high-energy electron diffraction and single wavelength ultra-fast ellipsometry (0.5 nm).

The magneto-optical transmission measurements were carried out by using a Fourier transform spectrometer coupled to a 16 T superconducting coil. The radiation of a Globar lamp is guided to a sample using oversized waveguides (light pipes). The intensity of the transmitted light is measured by a silicon bolometer. Both the magnet and the bolometer require cryogenic temperatures; therefore, most of reported up to today experiments were conducted at 4.2 K or lower temperatures. In this work, to perform a temperature tuning of the band structure, the standard magneto-optical configuration required important modifications. The bolometer was placed in a vacuum chamber separated from the sample chamber. To provide a wide spectral range for experiments, an indium-sealed cold diamond window ensures the optical coupling between the transmitted light and the bolometer. An additional superconducting coil around the bolometer compensates the spread field of the main coil, keeping the bolometer at zero magnetic field. This additional compensating superconducting coil also provides an additional screening of the bolometer. A Lambda plate coil—placed below the main magnet allows to obtain superfluid helium around the bolometer and keep the main coil at 4 K. Therefore, this modified experimental set-up allows to keep the coils in their superconducting state, the bolometer at its optimal temperature and to tune the sample chamber temperature in the 2–150 K range.

The magneto-optical spectra were measured in the Faraday configuration up to 16 T, with a spectral resolution of 0.5 meV. All the spectra were normalized by the sample transmission response at *B*=0 T.

### Simplified Kane model

Relativistic fermions are usually described by the Dirac equation:


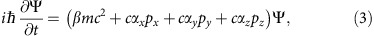


where *p*_*i*_ (*i*=*x*, *y*, *z*) are the components of momentum operator, *m* and *c* are the rest mass and velocity of light, respectively. The matrices *α*_*i*_ and *β* define the symmetry properties of the particles and have the form


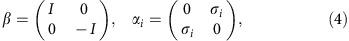


in which *σ*_*i*_ are the Pauli matrices and *I* is a 2 × 2 unit matrix. As it is clearly seen from equation (3), if the rest mass of particles equals zero, their dispersion is described by twofold degenerate cone in energy-momentum space.

Current physics has proven the existence of several bulk condensed-matter materials, which are fairly well described by the above equation. At the same time, there are also other systems with relativistic-like charge carriers; nevertheless, they are described by different Hamiltonians. For Kane fermions, the corresponding Hamiltonian 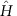 formally resembles the one for genuine 3D Dirac particles. However, we see that it has a form of a 6 × 6 matrix, which describes qualitatively a different system:


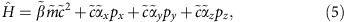


where 
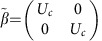
, 
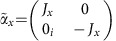
, 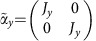, 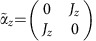 and where *U*_*c*_, *J*_*x*_, *J*_*y*_, *J*_*z*_ are 3 × 3 matrices described as follows:


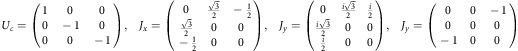


Here we deliberately present low-energy Hamiltonian, which describes the Kane fermions in a form similar to the Dirac equation, defining the rest mass 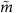 and velocity 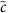 of the Kane fermions. The matrices *J*_*x*_, *J*_*y*_, *J*_*z*_ arising in equation (5) do not satisfy the algebra of angular momentum 1; therefore, the Hamiltonian 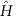 does not reduce to any well-known case of relativistic particles. However, the Kane fermions, with the Hamiltonian described by equation (5), share a number of properties with other relativistic particles.

### Data availability

The data that support the findings of this study are available from the corresponding authors on request.

## Additional information

**How to cite this article:** Teppe, F. *et al*. Temperature-driven massless Kane fermions in HgCdTe crystals. *Nat. Commun.* 7:12576 doi: 10.1038/ncomms12576 (2016).

## Supplementary Material

Supplementary Information Supplementary Figures 1-5, Supplementary Notes 1-2 and Supplementary References
