# Supplementary material for: Temperature-driven massless Kane fermions in HgCdTe crystals
Source: Nat Commun. 2016 Aug 30;7:12576. doi: 10.1038/ncomms12576 (PMC5013552; doi:10.1038/ncomms12576)
Supplement: Supplementary Information — Supplementary Figures 1-5, Supplementary Notes 1-2 and Supplementary References [file ncomms12576-s1.pdf]

Supplementary Figures

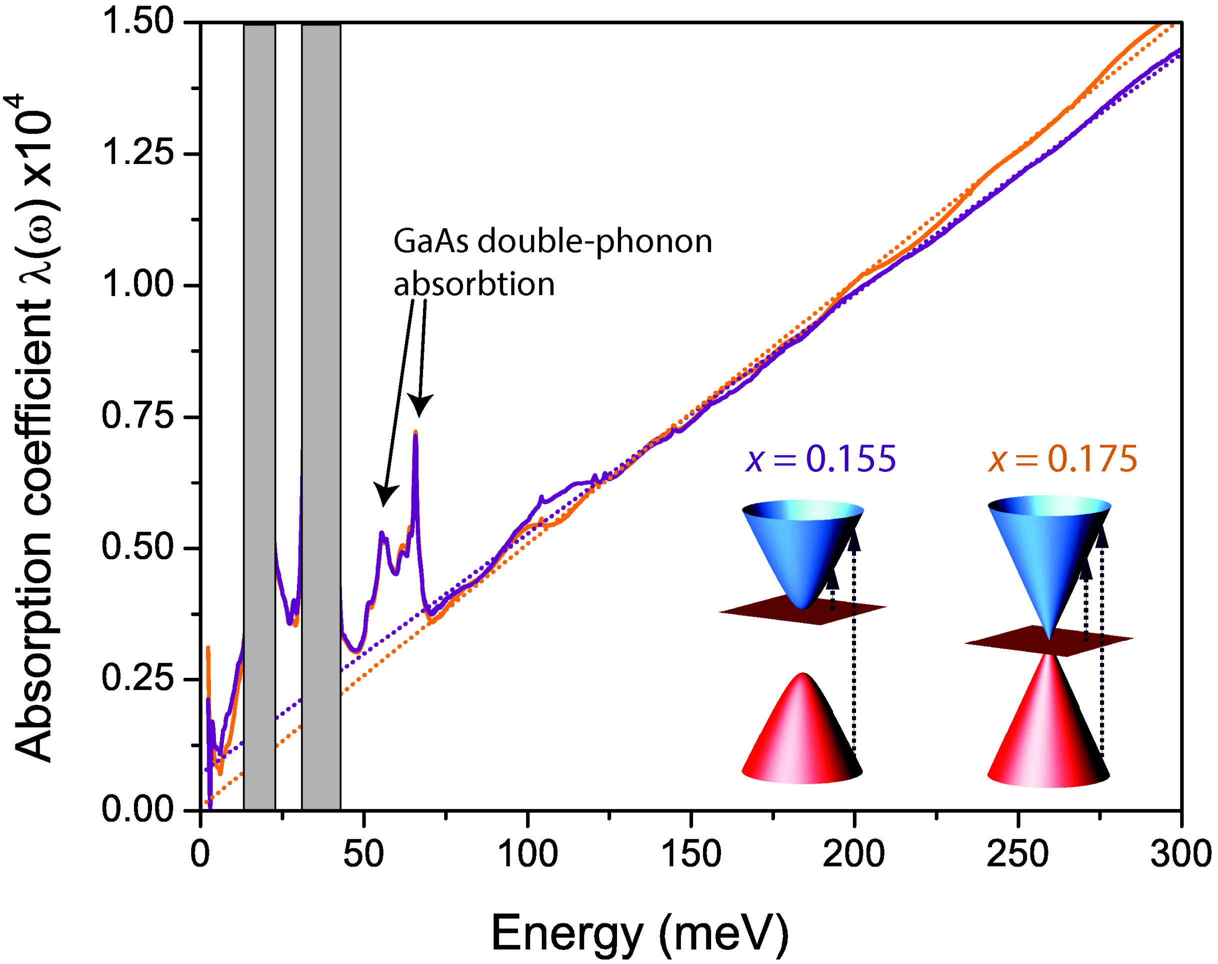

Supplementary Figure 1: Optical absorption of pseudo-relativistic Kane fermions in $Hg_{1-x}Cd_xTe$ at 4.2K. Zero field absorption coefficients exhibit a linear behavior reflecting the relativistic character of the 3D Kane fermions in $Hg_{1-x}Cd_xTe$. The band gap values of $2\widetilde{m}\tilde{c}^2$ equaling (4 ± 2) meV and (20 ± 4) meV for $x = 0.175$ and $x = 0.155$, respectively, are extracted from fits (dashed lines) based on Supplementary Equation (5) in Supplementary Note 2. The inset cartoon depicts inter-band transitions that contribute to the linear optical absorption.

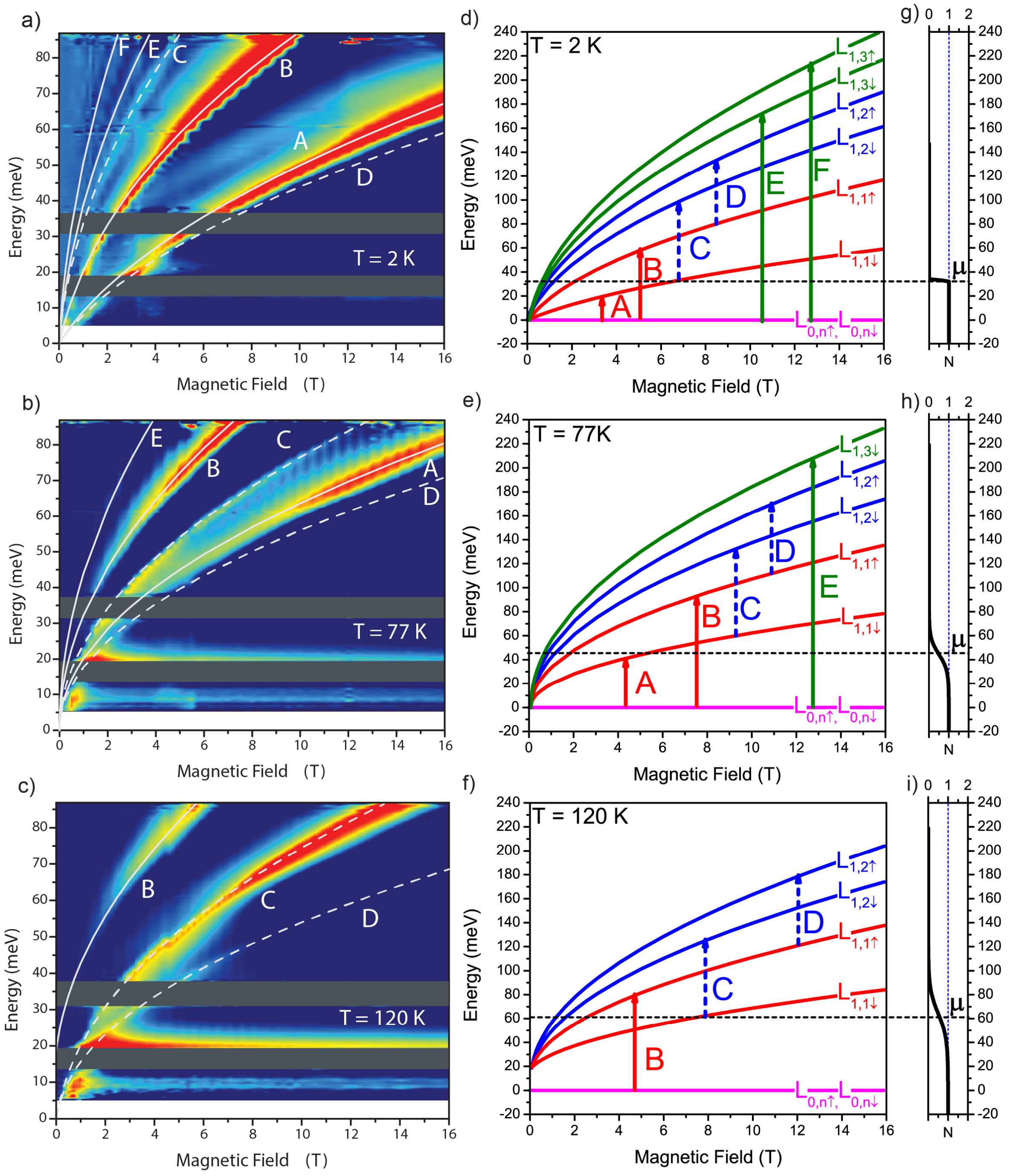

Supplementary Figure 2: Relative change of absorbance in sample B and Landau-level fan chart.
The inter-Landau-levels transitions visible in the color maps on panels **a)**, **b)**, and **c)** are plotted in Landau level fan chart on panels **d)**, **e)** and **f)**, respectively, and iterated with capital letters (A, B, C, D, E, F) for clarity. Three different charts are presented for the three different states, inverted in panels **a)** and **d)**, gapless in panels **b)** and **e)**, and normal band structure in panels **c)** and **e)** of HgCdTe compounds as a function of magnetic field, calculated using the simplified Kane model for x = 0.155 and T = 2 K, 77 K, and 120 K, respectively. Arrows show the optically allowed transitions ($L_{\xi,n,\sigma} \rightarrow L_{1,n+1,\sigma}$ with ξ=0,1 and n=0,2,3) observed in our magneto-transmission spectra. The horizontal black dashed line in **d)**, **e)** and **f)**, represents the chemical potential μ at zero magnetic field, calculated with the Supplementary Equations (2) to (4) in Supplementary Note 1, in which the electron concentration *N* was measured experimentally. The corresponding carrier distribution functions calculated for the three temperatures are shown on panels **g)**, **h)** and **i)**. The vertical blue dashed lines represent the position of the chemical potential.

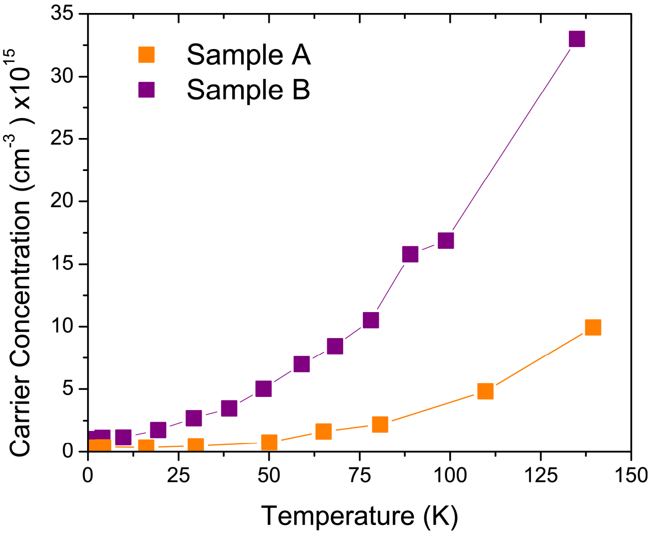

Supplementary Figure 3: The electron concentration as a function of temperature.
Measurements were performed by magneto-transport experiments. The values for 2 K, 77 K and 120 K were used to calculate the chemical potential μ within the Landau levels at those temperatures.

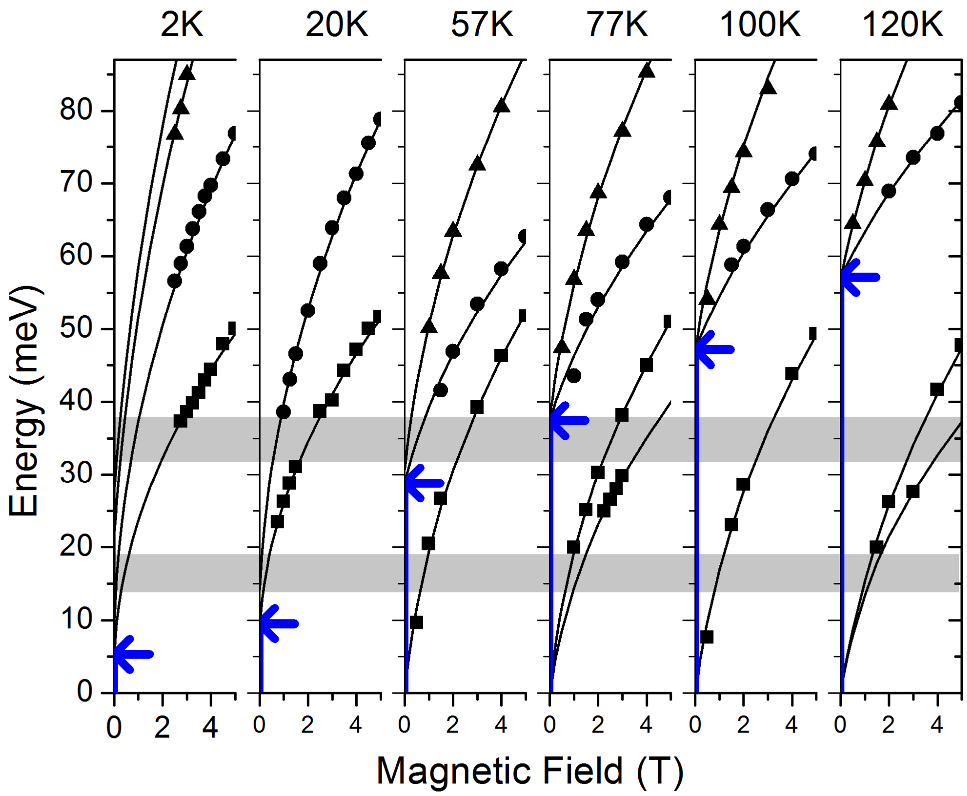

Supplementary Figure 4: The band gap evolution as a function of temperature for sample A.
Open symbols represent inter-band transitions and solid lines represent fits using simplified Kane model. The fitted lines coincide at zero magnetic field and point to a bandgap value, which increases with temperature from 5 meV up to 56 meV. Small full symbols are used to represent the intra-band transitions. Shaded areas represent the Reststrahlen bands. The behavior of the inter-band transitions at small magnetic fields gives information about the band gap, which is marked with blue arrows for every temperatures: 2 K, 20 K, 57 K, 77 K, 100 K and 120 K.

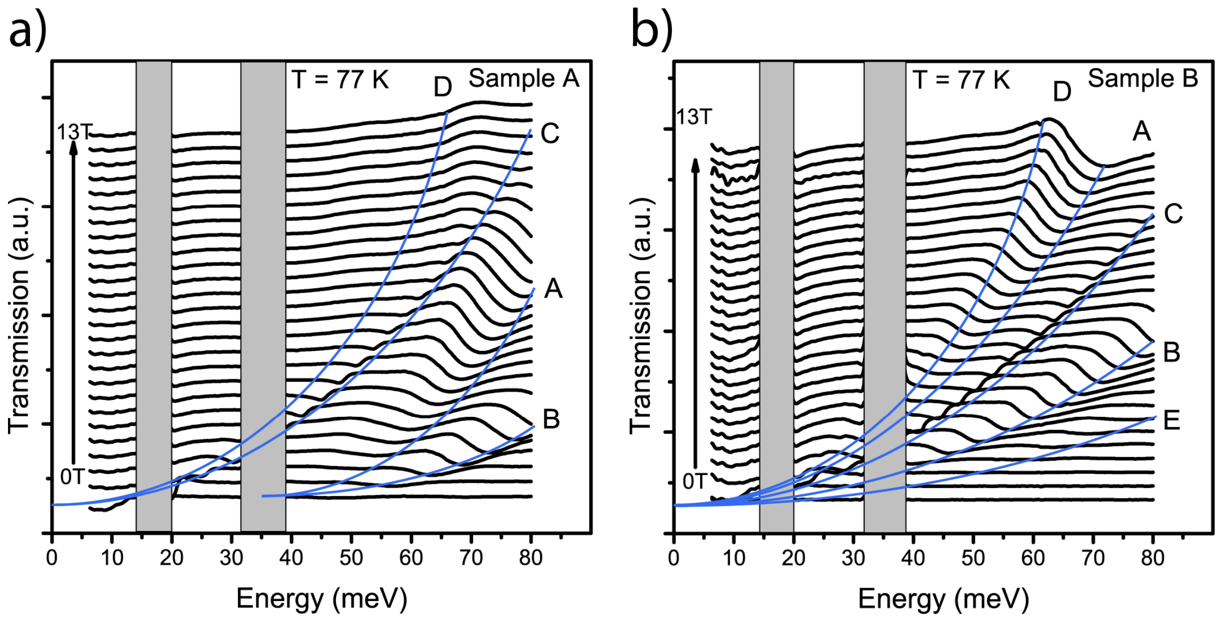

Supplementary Figure 5. Experimentally measured magneto-absorption spectra for samples A and B. Data for sample A and B are displayed in panels **a)** and **b)** respectively. Examples of transmission spectra measured at 77 K are shown as a waterfall plot for different magnetic field values up to 13 T with a 0.5 T resolution. Absorption lines are highlighted by placing a blue line in their vicinity and a letter allowing for the identification of given transitions. Transition A, B, C and D correspond to $L_{0,0\downarrow}\rightarrow L_{1,1\downarrow}$, $L_{0,0\uparrow}\rightarrow L_{1,1\uparrow}$, $L_{1,1\downarrow}\rightarrow L_{1,2\downarrow}$, $L_{1,1\uparrow}\rightarrow L_{1,2\uparrow}$ respectively. Transitions marked as D are almost invisible in that representation, blue lines mark their known positions. A gap of 36 meV is clearly visible in sample A, while the gapless state is undeniable in sample B at the same temperature.

Supplementary Notes

Supplementary Note 1: Temperature effects

The band-gap energy versus temperature is given by the empirical formula found in[1]:

$$E_g(eV) = -0.303(1-x) + 1.606x - 0.132x(1-x) + \frac{[6.3(1-x) - 3.25x - 5.92x(1-x)]10^{-4}T^2}{[11(1-x) + 78.7x + T]} \quad (1)$$

Color maps of the inter-Landau-level transitions in the sample B at temperatures from 2 K to 120 K are shown in the linear scale, in Supplementary Fig. 2 panels a), b) and c). Corresponding Landau-level fan chart emphasizing the observed optical transitions, are given for each temperature in panels d), e) and f). The calculated chemical potential is presented in panels h), i) and j). Its position allows one to evaluate the energy range at which optical transitions can take place.

To find the distribution of occupied electronic states in the conduction band at given temperature, one needs to calculate density of states $D(E)$ and total chemical potential μ for the sample. Starting from expression (1) in the main text for the band dispersion, we arrive to the following expression for $D(E)$:

$$D(E) = \frac{1}{\pi^2 c^3 \hbar^3}(E - \tilde{m}\tilde{c}^2)\sqrt{(E - \tilde{m}\tilde{c}^2)^2 - \tilde{m}^2\tilde{c}^4} \cdot \theta(E - \tilde{m}\tilde{c}^2) \quad (2)$$

By using experimental values of $\tilde{m}$, $\tilde{c}$ and electron concentration N, total chemical potential μ, which defines the distribution of occupied electronic states, can be obtain by solving the following integral equation

$$N = \int_{\tilde{m}\tilde{c}^2}^{+\infty} f(E,\mu,T) D(E) dE, \quad (3)$$

where $f(E, \mu, T)$ is a Fermi-Dirac distribution function

$$f(E,\mu,T) = \frac{1}{\exp\left(\frac{E-\mu}{k_B T}\right)+1}. \quad (4)$$

Here k_B is the Boltzmann constant.

The electron concentration N was experimentally determined as a function of temperature for both samples A and B by Hall measurements. These results allowing for the calculation of the chemical potential μ, are shown in Supplementary Fig. 3.
The transmission spectra of both samples at T = 77 K, for magnetic field values up to 13 T, are shown in Supplementary Fig. 5. Convincingly, sample A exhibits a band gap of 36 meV while sample B is gapless at the same temperature of 77 K.

The positions of the minima in transmission spectra are plotted by symbols as a function of magnetic field for temperatures from 2 K to 120 K in Supplementary Fig. 4. The inter-band transitions, which give the band gap value at zero magnetic field, are represented by open symbols while intra-band transitions are shown by small full symbols. The solid lines are theoretical curves obtained by using the simplified Kane model. One can clearly see the opening of the band gap and the reliable determination of its energy during temperature increase.

Supplementary Note 2: Zero magnetic field optical conductivity

Due to the conical dispersion of the massless Kane fermions in HgCdTe crystals, absorption coefficient λ of both samples measured at 4.2 K exhibits a linear behavior as a function of photon energy $\hbar\omega$ at high energies similar to 3D Weyl systems[2]. This linear behavior of the optical conductivity provides a clear evidence for the pseudo-relativistic behavior of the 3D particles in any Dirac/Weyl-like materials, as discussed by Timusk[3]. However, if the HgCdTe layer is not exactly gapless, the hyperbolic dispersions mimic the linear energy-momentum law at higher energies, exceeding the band gap values. The relatively simple form of eigenvalues of the Kane fermion's

Hamiltonian in equation (1) in the main text allows one to calculate the absorption coefficient above $2\widetilde{m}\widetilde{c}^2$ as a function of $\hbar\omega$ analytically[4]:

$$\lambda(\hbar\omega) = \frac{K}{(\hbar\omega)^3}\left[(1-\eta)\sqrt{(1-\eta)^2 - \eta^2} + \frac{1}{8}\sqrt{1-4\eta^2}\right], \qquad (5)$$

where $\eta = \widetilde{m}\widetilde{c}^2/\hbar\omega$ and K are constants. The first term in brackets describes absorption coefficient involving flat (heavy-hole) band, while the second term corresponds to the contribution of the light hole band. By analyzing the absorption coefficient with Supplementary Equation (5) at energies exceeding the GaAs substrate Reststrahlen band, shown in the Supplementary Fig. 1, we extract the band gap values $2\widetilde{m}\widetilde{c}^2$ at low temperatures for both samples, which are approximately 4 ± 2 meV and -20 ± 4 meV for the samples A and B, respectively. As it is for Dirac fermions obeying equation (3) of the main text, the appearance of non-zero band gap does not exclude pseudo-relativistic character of the Kane fermions, which is retained at energies significantly above (or below) $2\widetilde{m}\widetilde{c}^2$. In our case, the presence of pseudo-relativistic 3D fermions is also revealed by the linear character of absorption coefficient as a function of $\hbar\omega$ at high energies.

Supplementary References

1. Laurenti, J. P., Camassel, J., Bouhemadou, A., Toulouse, B., Legros, R., and Lusson, A. et al. Temperature dependence of the fundamental absorption edge of mercury cadmium telluride. *J. Appl. Phys.* 67, 6454-6460 (1990).
2. Malcolm, J. D. and Nicol, E. J. Magneto-optics of massless Kane fermions: Role of the flat band and unusual Berry phase. *Phys. Rev. B* 92, 035118 (2015).
3. Timusk, T., Carbotte, J. P., Homes, C. C., Basov, D. N. and Sharapov, S. G. Three-dimensional Dirac fermions in quasicrystals as seen via optical conductivity. *Phys. Rev. B* 87, 235121 (2013).
4. Chang, Y., Grein, C.H., Sivananthan, S., Flatte, M.E., Nathan, V., and Guha, S. Narrow gap HgCdTe absorption behavior near the band edge including nonparabolicity and the Urbach tail. *Appl. Phys. Lett.* **89**, 062109 (2006).
